# Oral Focal Mucinosis in a Patient From Hail, Saudi Arabia: A Case Report of a Rare Gingival Lesion

**DOI:** 10.7759/cureus.112036

**Published:** 2026-07-04

**Authors:** Fahad Yousef Alghaithi, Mohammed Aljezoli, Abdulrahman M Alanazi

**Affiliations:** 1 College of Dentistry, University of Ha'il, Hail, SAU; 2 Department of Oral and Maxillofacial Surgery, King Khalid Hospital, Hail, SAU

**Keywords:** excision of tumour, gingival tumor, maxilla mass, mucosal biopsy, oral focal mucinosis

## Abstract

Oral focal mucinosis (OFM) is an uncommon, benign condition of the oral cavity marked by localized myxoid degeneration of the connective tissue. This report presents a highly unusual case of a large OFM localized to the maxillary gingiva of a 55-year-old female patient in Hail, Saudi Arabia. Because its clinical appearance closely mimics reactive overgrowths like pyogenic granuloma, establishing a clinical diagnosis is difficult. Consequently, definitive diagnosis relies entirely on histopathologic assessment. While gingival OFM remains exceedingly rare, it should be routinely included in the differential diagnosis of exophytic oral masses.

## Introduction

Oral focal mucinosis (OFM) is an uncommon, benign mucosal lesion characterized by localized myxoid degeneration of the oral connective tissue, resulting from an overproduction of hyaluronic acid by fibroblasts, which was first recognized and described as a distinct clinicopathologic entity by Tomich in 1974 [1]. Epidemiologically, the lesion displays a strong female-to-male predilection, with the gingiva serving as the most common intraoral site, accounting for up to 75% of all documented cases [2]. Clinically, OFM typically presents as a slow-growing, painless, exophytic nodule matching the color of the adjacent mucosa [3]. Because its clinical features are highly non-specific and lack distinctive diagnostic markers, OFM frequently misleads clinicians by perfectly mimicking more common reactive soft-tissue overgrowths, such as pyogenic granulomas, peripheral giant cell granulomas, or peripheral ossifying fibromas (POFs) [4].

Establishing a precise preoperative diagnosis based purely on clinical visual inspection is notoriously difficult, making definitive histopathological analysis completely mandatory [5]. Microscopically, the lesion is confirmed by identifying a well-localized, non-encapsulated zone of loose, myxomatous stroma embedded with stellate or spindle-shaped fibroblasts that characteristically lack prominent vascular proliferation or significant inflammatory infiltrates [2]. While conservative local surgical excision is curative with exceptionally low recurrence rates, documented reports of OFM within Saudi Arabia remain exceedingly rare [5]. This report describes a rare case of a large, exophytic OFM of the maxillary gingiva in a 55-year-old female patient from Hail, highlighting the clinical challenges and the crucial role of microscopic evaluation.

## Case presentation

**Figure 1:**
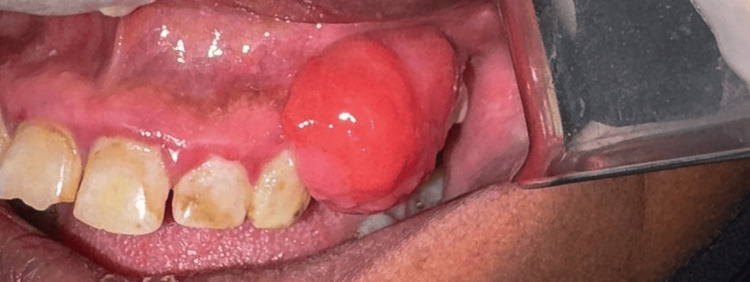
Preoperative intraoral view showing a large, lobulated, exophytic soft tissue mass on the left maxillary gingiva. The lesion originates from the attached gingiva and interdental papilla, extending from tooth 23 to tooth 26. The exophytic mass measures approximately 2.2 cm by 1.8 cm in its greatest dimensions. The lesion was initially clinically suspected to be a reactive overgrowth due to local plaque accumulation.

A 55-year-old female patient presented with a chief complaint of an uncomfortable overgrowth of tissue in the upper left gingiva. A large, mobile, pedunculated exophytic mass was present on the attached gingiva and interdental papilla of the upper left quadrant, extending from the distal aspect of the canine (tooth 23) to the mesial aspect of the first molar (tooth 26) (Figure 1).

The patient reported that this lesion had first appeared approximately one year ago and had gradually increased in size, causing mild local discomfort and functional interference, though it otherwise remained asymptomatic.

The lesion exhibited a dusky red coloration and a soft-to-firm consistency with normal, non-ulcerated overlying mucosa. There was no history of trauma, no pathologic mobility of the adjacent teeth was noted, and the patient presented with poor oral hygiene. The patient's medical history revealed well-controlled type 2 diabetes mellitus and hypertension, for which she takes regular prescribed medications. She reported no history of drug allergies within the past six months and was not pregnant.

Based on the clinical presentation and poor oral hygiene, a provisional diagnosis of a localized reactive gingival overgrowth was made. The primary differential clinical diagnoses considered were pyogenic granuloma, peripheral giant cell granuloma (PGCG), and POF. Under local anesthesia, a complete conservative excisional biopsy was performed using a #15 surgical blade. The lesion was carefully grasped with tissue forceps and excised cleanly from its base.

**Figure 2:**
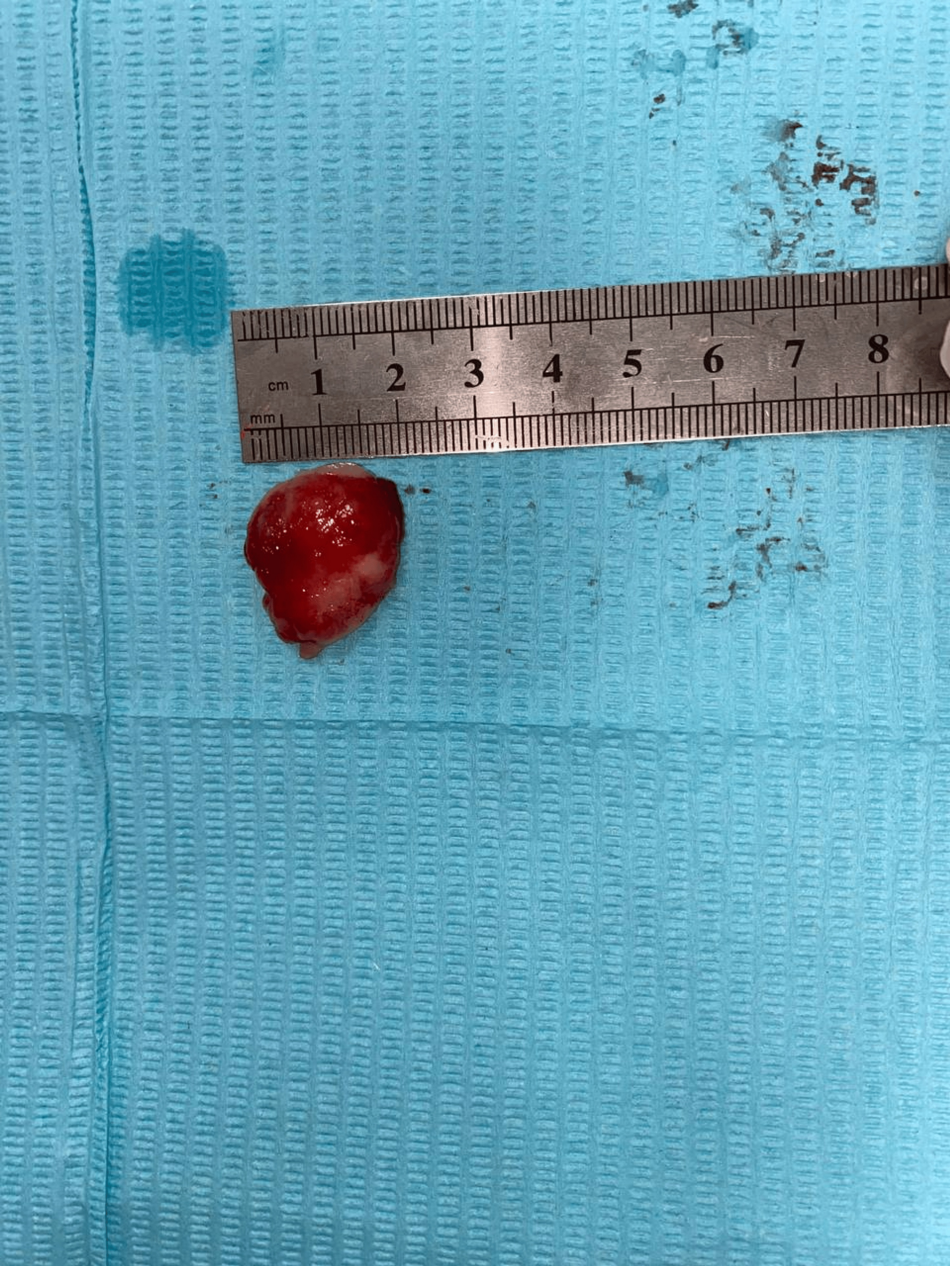
Measurement of the horizontal width demonstrating a size of approximately 1.8 cm.

**Figure 3:**
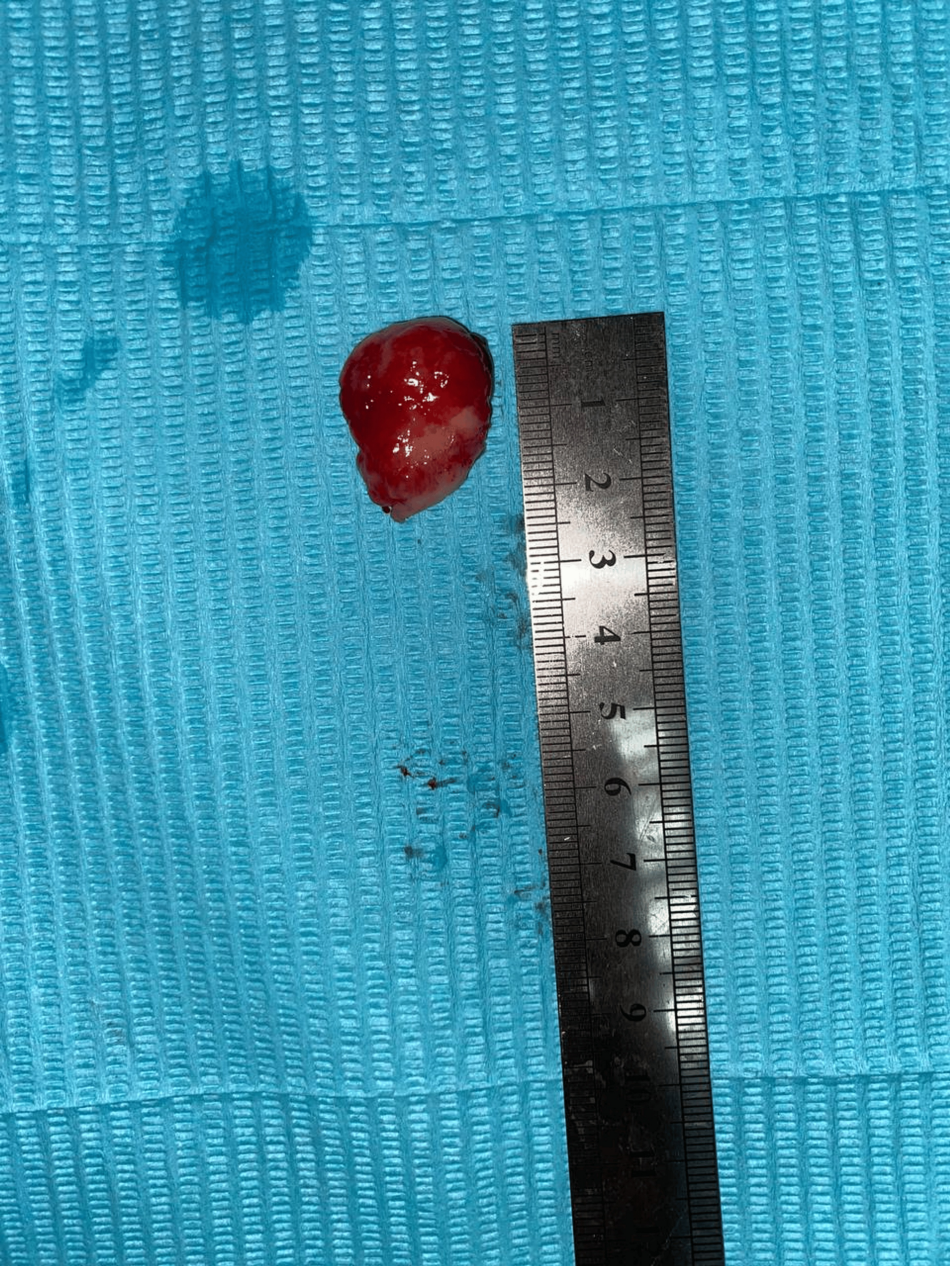
Measurement of the vertical length confirming a size of approximately 2.2 cm.

Following removal, thorough curettage of the surgical site was performed, and the area was meticulously irrigated with sterile 0.9% normal saline. Immediate post-operative hemostasis was achieved via gauze compression. The excised soft tissue specimen confirmed the final dimensions of 2.2 cm in length and 1.8 cm in width (Figure 2 and Figure 3).

**Figure 4:**
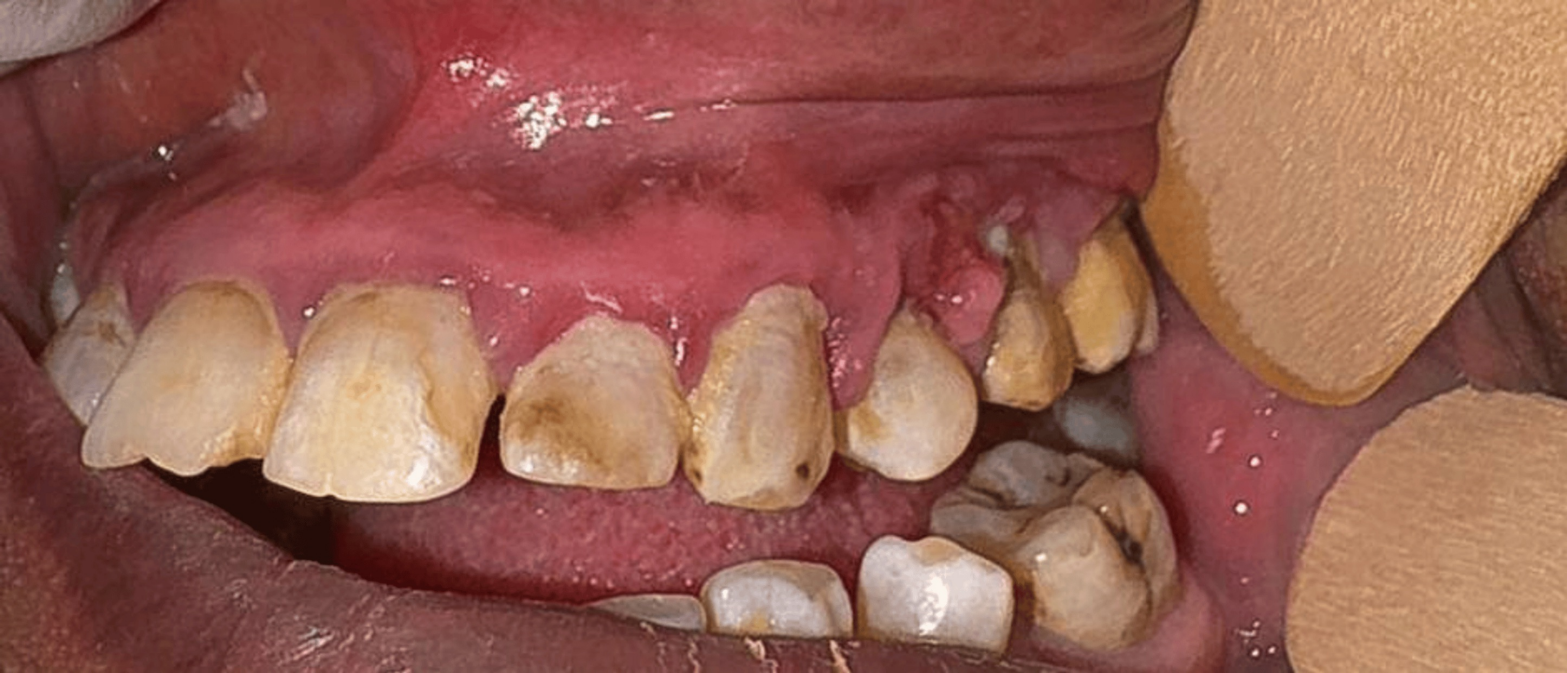
Postoperative intraoral view taken seven days following the excisional biopsy, demonstrating excellent primary healing of the maxillary gingival mucosa.

The surgical specimen was placed in a container filled with 10% formalin and submitted for histopathological evaluation. The patient was given comprehensive post-operative instructions and prescribed systemic antibiotics (Amoxicillin/Clavulanate (Augmentin) 1 g twice daily for seven days) along with an analgesic (Paracetamol 500 mg three times daily as needed for pain). The surgical wound healed satisfactorily without any immediate complications (Figure 4).

To date, the patient remains under regular clinical recall, showing complete restoration of the gingival architecture with no clinical evidence of recurrence. Written informed consent was obtained from the patient to publish this case report and any accompanying clinical images. 

**Figure 5:**
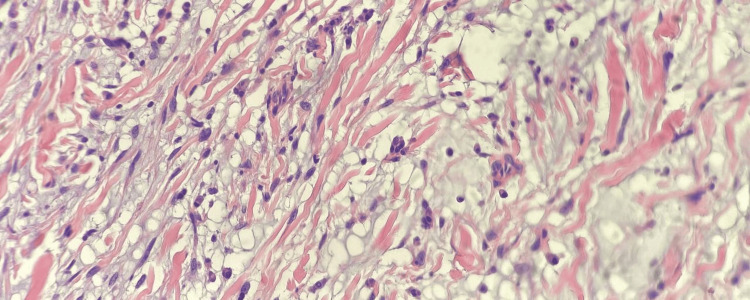
Histopathological examination of the biopsy specimen via hematoxylin and eosin (H&E) staining (20 times magnification), confirming the diagnosis of oral focal mucinosis, characterized by an unencapsulated myxomatous stroma.

Histopathological examination revealed a lesion beneath the surface stratified squamous epithelium composed of scattered stellate cells in a myxoid connective tissue background and surrounding fibrocollagenous tissue. The surrounding connective tissue shows a prominent mixed inflammatory infiltrate, predominantly plasma cells, and perivascular lymphocytic infiltrate. No giant cell identified. No dystrophic calcification, lamellar or woven bone present (Figure 5). 

## Discussion

OFM is a rare, benign soft-tissue condition that poses an immediate diagnostic trap for the clinical oral surgeon. Since its initial description by Tomich [1], epidemiologic data have established a strong female predilection, typically presenting in the fourth and fifth decades of life [2]. This classic demographic profile matches our 55-year-old female patient perfectly. However, while the literature describes the typical OFM lesion as a small, pinkish, asymptomatic nodule under 2 cm [2,3], our case presented an unusually aggressive clinical footprint measuring 2.2 × 1.8 cm. The lesion in this patient was a large, mobile, exophytic mass that spanned across almost an entire maxillary quadrant, extending mesiodistally from the canine (23) all the way to the first molar (26).

This substantial size, combined with a distinctly dusky red color and the patient's poor baseline oral hygiene, heavily favored a provisional diagnosis of a chronic reactive soft-tissue lesion over a rare idiopathic condition. Local irritants like plaque and calculus accumulation are well-documented triggers for rapid tissue proliferation in the gingiva [5,6]. Consequently, pyogenic granuloma, PGCG, and POF were considered our primary clinical differentials.

A pyogenic granuloma was highly suspected due to the dusky red coloration, though it typically exhibits a severe tendency to bleed during examination, a feature notably absent in this case [2]. PGCG was another strong possibility given the exophytic morphology on the attached gingiva, but it often manifests with a deeper blue-purple hue and can show a characteristic cupping resorption of the underlying alveolar bone on radiographs, which our patient did not exhibit [7]. A POF was also considered, but these lesions typically display a much firmer consistency upon palpation due to internal calcifications, contrasting with the soft-to-firm, highly mobile nature of the mass we excised [8].

Furthermore, establishing a definitive diagnosis of OFM requires a meticulous histopathological differentiation from other true intraoral myxoid neoplasms and lesions [2]. Odontogenic myxoma must be excluded; while it presents with a similar myxoid stroma, it is primarily an intraosseous bone-destructive neoplasm containing odontogenic epithelial rests, unlike the purely peripheral soft-tissue nature of OFM [2]. Neurogenic myxoid tumors, such as nerve sheath myxoma and myxoid neurofibroma, are differentiated by their distinct lobular architecture and strong immunohistochemical positivity for S-100 protein [2]. True soft-tissue myxomas and myxofibromas exhibit greater cellular atypia, pleomorphism, and a higher vascular framework compared to the completely benign, hypocellular, and avascular myxoid core that defines true OFM [2].

Because a definitive diagnosis cannot be safely established through visual inspection or clinical palpation alone, microscopic evaluation remains the undisputed gold standard for these lesions [4]. Histopathologically, OFM is characterized by a localized, unencapsulated pool of loose, myxomatous connective tissue containing spindle- or stellate-shaped fibroblasts [2,4]. Crucially, it lacks the dense capillary proliferation seen in pyogenic granulomas and the abundant multinucleated giant cells that define a PGCG [2]. Furthermore, while an ordinary irritation fibroma can occasionally undergo focal myxoid degeneration, it is easily differentiated from OFM because its myxoid changes are strictly localized, whereas true OFM consists of an extensive, uniform myxoid stroma surrounded by normal dense collagen [2,4]. While classic OFM typically demonstrates a minimal inflammatory infiltrate, the presence of localized plasma cells and lymphocytes in our case can be attributed to secondary chronic inflammation induced by the patient's poor oral hygiene and persistent plaque accumulation on the gingival margin.

A limitation of this case report is the lack of embedded preoperative periapical or panoramic radiographs within the documentation; however, clinical and intraoperative evaluation confirmed a completely peripheral lesion with zero bony involvement or alveolar bone resorption.

Our management of this case followed classic oral surgery protocols via a definitive excisional biopsy under local anesthesia [9]. Using a #15 surgical blade, a clean incision was made at the base of the pedunculated mass to ensure complete removal. Complete excision is recommended in OFM management, as recurrence has been reported mainly in association with incomplete removal [9,10]. Local curettage of the underlying gingival site was performed, followed by sterile saline irrigation. Post-operative management with a seven-day course of Augmentin (1 g BID) and Paracetamol (500 mg PRN) resulted in an uneventful, satisfactory healing phase.

To investigate the prevalence of this condition, a comprehensive literature search was conducted across major electronic databases, including PubMed, Google Scholar, and ScienceDirect, utilizing keywords such as “oral focal mucinosis”, “gingival myxoma”, and “Saudi Arabia”. Based on this verified database screening, documented cases of OFM within the Kingdom remain exceptionally rare and are primarily confined to major tertiary academic centers. To the best of our knowledge, this represents the first documented clinical case of OFM originating specifically in Hail, Saudi Arabia. This regional scarcity highlights the importance of reporting this case to expand the geographical and epidemiological mapping of rare oral pathologies within the Kingdom. This case highlights that OFM can present as a remarkably large, dusky red mass mimicking standard reactive lesions, especially in the presence of poor oral hygiene. Clinicians operating within regional departments must maintain a high index of suspicion and rely firmly on histopathological confirmation when evaluating atypical exophytic growths of the gingiva.

## Conclusions

Although OFM remains an exceedingly rare benign entity, it must be included in the differential diagnosis of large, exophytic masses involving the gingiva, particularly when local clinical features and poor oral hygiene strongly mimic chronic reactive overgrowths. Because it has no pathognomonic clinical or radiographic markers, accurate diagnosis cannot be established through visual inspection alone, making definitive histopathological evaluation the undisputed gold standard to prevent misdiagnosis. Complete conservative surgical excision down to the underlying bone remains the treatment of choice. While our case represents a unique regional presentation within Hail, providing essential data to the oral surgery and pathology literature in Saudi Arabia, conclusions regarding its definitive long-term behavior remain limited by a short tracking period. Therefore, establishing a systematic, longitudinal follow-up protocol is essential to thoroughly monitor and record any potential long-term recurrence patterns in clinical practice.
